# A New Paradigm for the Study of Cognitive Flexibility in Children and Adolescents: The “Virtual House Locomotor Maze” (VHLM)

**DOI:** 10.3389/fpsyt.2021.708378

**Published:** 2021-09-23

**Authors:** Alexander Castilla, Gregoire Borst, David Cohen, Jacques Fradin, Camille Lefrançois, Olivier Houdé, Mohamed Zaoui, Alain Berthoz

**Affiliations:** ^1^Université de Paris, LaPsyDÉ, CNRS, Paris, France; ^2^Laboratoire de Psychologie et de Neurosciences, Institut de Médecine Environnementale (IME), Paris, France; ^3^Centre Interdisciplinaire de recherche en Biologie (CIRB), Collège de France, Paris, France; ^4^Institut Universitaire de France (IUF), Paris, France; ^5^Département de Psychiatrie de l'Enfant et de l'Adolescent, AP-HP, Hôpital Pitié-Salpêtrière, and Institut des Systèmes Intelligents et de Robotiques, Sorbonne Université, Paris, France

**Keywords:** replanning, spatial navigation, executive functions, cognitive control, locomotor protocol, visuospatial abilities

## Abstract

Classical neuropsychological assessments are designed to explore cognitive brain functions using paper-and-pencil or digital tests. The purpose of this study was to design and to test a new protocol named the “Virtual House Locomotor Maze” (VHLM) for studying inhibitory control as well as mental flexibility using a visuo-spatial locomotor memory test. The VHLM is a simple maze including six houses using the technology of the Virtual Carpet Paradigm*™*. Ten typical development children (TD) were enrolled in this study. The participants were instructed to reach a target house as quickly as possible and to bear in mind the experimental instructions. We examined their planning and replanning abilities to take the shortest path to reach a target house. In order to study the cognitive processes during navigation, we implemented a spatio-temporal index based on the measure of kinematics behaviors (i.e., trajectories, tangential velocity and head direction). Replanning was tested by first repeating a path chosen by the subject to reach a given house. After learning this path, it was blocked imposing that the subject inhibited the learned trajectory and designed a new trajectory to reach the same house. We measured the latency of the departure after the presentation of each house and the initial direction of the trajectory. The results suggest that several strategies are used by the subjects for replanning and our measures could be used as an index of impulsivity.

## Introduction

The human brain combines many cognitive processes to understand the world around us and to enable us to adapt to different situations in a vicarious way (i.e., brain capacity for finding different solutions for a given problem) (1). This adaptation requires that learned associations to solve specific problems are sometimes challenged by new events needing different strategies. Spatial navigation abilities such as planning, replanning, visuo-spatial working memory and inhibition are crucial for everyday activity. For instance, inhibitory mechanisms allow the brain to perform these changes in strategies and to develop in children during ontogeny (2–4). Inhibitory processes can be observed during the very early timing for the initiation of an action, or when a re-planning is required. In that case, a delay is caused by the time necessary for the brain to (a) inhibit the learned process, and (b) elaborate the new strategy (5–7). Numerous psychological and neuropsychological tasks using paper-and-pencil assessment have documented this cognitive delay. We suppose here that such a delay also appears during a spatial navigation task in which subjects must re-organize a memorized locomotor trajectory.

Throughout typical cognitive development (TD), inhibitory control is actively involved during selective attention when irrelevant information needs to be ignored or inhibited, and during the production of optimal behavioral response (e.g., stop an action or avoiding reacting). Children are always confronted to a choice between using automatic strategies (i.e., intuitive system or so called “heuristics”), or substituting strategies based on logical algorithms (i.e., analytical system) in order to accomplish a goal in specific contexts (3, 8, 9). Concerning neurodevelopmental disorders, the inhibition process is particularly affected in the attention deficit disorder with or without hyperactivity (ADHD) that is characterized by above-normal levels of hyperactive and impulsive behaviors and below-normal levels of attention (DSM-V) (10). In ADHD, attention deficit and impulsiveness due to poor inhibition processes can lead to academic learning impairments (11) and difficulties in planning adequate behavior (12).

Previc (13) puts forward a neuropsychological model based on the egocentric space segmentation linked with the motor capacities and perceptual integration. His model proposes a division of space in four key behavioral “realms” in addition to body space: the peripersonal space (PPS) (i.e., the reaching space), the focal extrapersonal space (FE), the action extrapersonal space (ES) and the ambient extrapersonal space (AES). This corresponds to what neurologists had identified long ago, from observing pathological deficits following brain lesions, as body space, peripersonal space, near distant action space and environmental action space (14, 15). The extrapersonal space serves to navigate and to orientate oneself toward objects that are situated beyond the near-body space. Recently, it has been suggested that this segmentation of space is based on a modular organization of the brain networks in which each type of action space requires specific geometries. These types of geometries are composed of Euclidean and Non-Euclidean geometries and they are regrouped in a general geometry called Topos (4, 16). The understanding of the different brain networks for different action spaces is an essential factor to consider during the assessment of spatial navigation. For instance, spatial navigation abilities have been extensively tested using the mobile-app-based cognitive task (Sea Hero Quest) assessing planning-replanning and inhibition (17). However, specialized cognitive navigational tasks involving the locomotor space could reveal complementary information concerning spatial navigation abilities. The present specifical designed protocol concerns only the “near distant extrapersonal locomotor space” which is described as walking around a room.

Piccardi et al. (18) suggested adapting a classical table spatial memory test, the table Corsi test, to the locomotor space, and proposing an initial version of the paradigm. They designed a test with tiles on the floor. The experimenter walks on these tiles in a sequence and asks the subject to observe, memorize and repeat the sequence by walking on the tiles. A span from one to nine tiles was used. Overall, normal subjects can successfully repeat up to five tiles, and exceptionally up to seven tiles depending on the difficulty of the path. This paradigm was called the Waking Corsi Test(WalCT). Subsequently, Alain Berthoz and his team developed the “Magic Carpet” which is a computerized version of the previous WalCT test. It uses translucent tiles equipped with lights for presenting the sequences and allowing the tactile sensors to record the timing. These flexible features provide accurate measurements of the subjects' performance. Several studies were conducted using this design on normal children and adults (19, 20). A difference in cognitive performance was observed in children and adults in the visuo-manual space (VMS) and visuo-locomotor space (VLS) (named by the authors “micro and macro spaces”).

By comparing the memory span in children using the walking Corsi test (WalCT) for the locomotor space vs the classical Corsi block tapping test (CBT) (21), the participants were asked to reproduce a series of sequences in a specific order. The results indicated that the youngest children presented no difference in the memory span in either test. However, a clear difference was observed from 5 to 6 years old characterized by a better performance in the classical Corsi block tapping test. The authors suggest that children develop spatial memory in the reaching space sooner than in the navigational space. In addition, the analysis of the performance in the WalCT revealed the existence of gender differences in spatial memory (22). Moreover, the adaptation of the classical cognitive test (i.e., Stroop Test) to spatial tasks facilitated the detection of an early cognitive impairment compared to the standard neuropsychological test (20, 23).

A similar study confirmed that the working memory span in the CBT is significantly greater than in the WalCT and that it improves in both tests during development (CBT and WalCT) (19). The results demonstrate an improved performance in boys aged 10 to 11 in the navigational space but found a significant distinction between the CBT and the WalCT. These findings provide clear indications that a cognitive performance is somehow modulated by space. In addition, it was used in children with Cerebral Palsy (24), and in adults with mild cognitive deficits (MCD), hippocampal lesions, cortical, or cerebellar deficits (20, 25).

From a scientific perspective of brain spatial modularity (i.e., reaching space and locomotor space) and given the lack of tools to explore human cognition during navigation, especially cognitive control and regarding that re-planning a learned task is an effective paradigm for studying executive functions and the role of inhibition during navigation. We devised a new protocol and a methodology to study behavioral indices of cognitive control during locomotion.

To our knowledge, no study has addressed the question of replanning and inhibition with a specifically designed protocol using a visuo-spatial locomotor memory test. The objective of the current study was to design and to test a new protocol named the “Virtual House Locomotor Maze” (VHLM). It allowed us to study cognitive control during navigation throughout neurocognitive development based on the Virtual Carpet paradigm. We explored the capacities of planning and replanning trajectories to study inhibition and mental flexibility using a spatio-temporal index by the means of measuring kinematic behavior (i.e., trajectories, tangential velocity and head direction) during spatial navigation. We concentrated our analysis on : (a) comparing the departure's latency (delays) of the overlearned paths and the new replanned path, (b) analyzing directional locomotor trajectories during replanning, and (c) exploring age and cognitive performance. This idea is built on the typical developmental studies based on the walking Corsi or Magic Carpet, which have shown that the development of spatial cognition plays an important role in the switching of cognitive strategies (26).

## Methods

### Participants

The experiment was carried out following the ethical standards established by the Declaration of Helsinki (27) and approved by Paris University's ethical committee (n° 2019-26-CASTILLA-COHEN). Ten children with typical development (TD) (six boys and four girls) aged from 9 to 16 years (mean = 10.72, SD = 2.45) were included in the pilot study (See Table 1). The parents or participant's legal representative signed an informed consent document before participating in the pilot study. Young participants gave their consent orally. Each participant was assessed individually. The study took place in the department of child and adolescent psychiatry at the Hospital Pitié Salpêtrière Paris, FranceAll TD children were relatives of the staff members of the department. All TD children had normal or corrected-to-normal vision and did not present any neurological or neuropsychological disorders nor exhibited any motor difficulties in gait.

**Table 1:** Subjects characteristics of the sample.

**Subject no**.	**Age (years)**	**Gender**	**Height (m)**	**Hand dominance**	**Mean tangential velocity (Cm/s)**
1	9	M	1.26	Right	90.2
2	16	M	1.70	Left	93.7
3	9	M	1.23	Right	91.9
4	9	F	1.11	Right	82.6
5	10	M	1.23	Right	78.6
6	10	F	1.48	Right	89.5
7	14	M	1.57	Right	81.9
8	9	F	1.06	Right	87.5
9	10	F	1.34	Right	79.5
10	9	M	1.10	Right	88.7

### Experimental Setup

#### The Virtual Carpet™ Paradigm

The experimental set-up used was the Virtual Carpet™ paradigm (24) that combines two computers, one video projector and one HTC Vive (HTC® Vive, Taiwan) for the experimentation. The first computer is connected to a video projector for projecting the navigational space and the stimuli over the floor. The second computer is connected with the HTC Vive and it runs the Basic Trajectory Software version 1 (BTS) to track the participant's trajectory and the navigational space's configuration.

**Figure 1:**
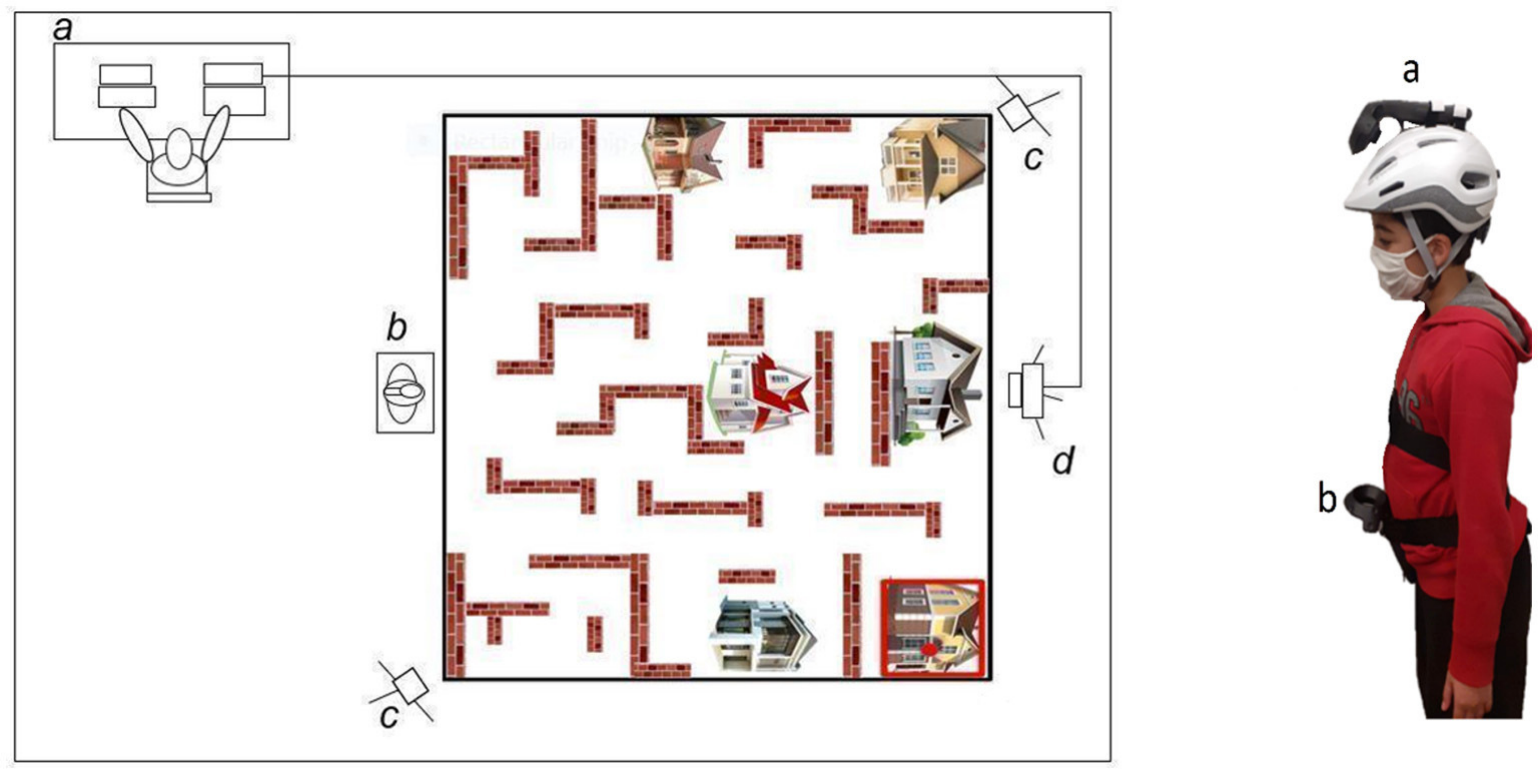
**(A)** The representation of the experimental setup: (a) control desk location where the experimenter runs the experiment, (b) departure point, (c) two HTC Vive cameras, (d) the video projector. **(B)** 3D motion sensors motion-tracker. (a) one handheld 3D motion sensor is adapted to a bike helmet and is worn on the participant's head and (b) the second handheld 3D motion sensor is attached to the belt which worn on the participant's waist.

The HTC Vive is a virtual reality system equipped with two infrared cameras, two handheld three-dimensional space (3D) motion sensors and one virtual reality headset. The two infrared cameras are placed 5 meters (16.4 feet) apart diagonally to cover the room in order to record the position of the two handheld 3D sensors (Figure 1A). The two handheld 3D sensors are used as motion-trackers during the experimentation. The first handheld 3D motion sensor (i.e., controller) is adapted to a bike helmet and is worn on the participant's head. The second handheld 3D is attached to the belt which is worn on the participant's waist (Figure 1B). Both motion sensors provide respectively 3D motion information from the head and the waist. The headset was only used during the calibration procedure to determine the navigational space in 3D coordinates system.

#### The Virtual House Locomotor Maze Protocol (VHLM™)

**Figure 2:**
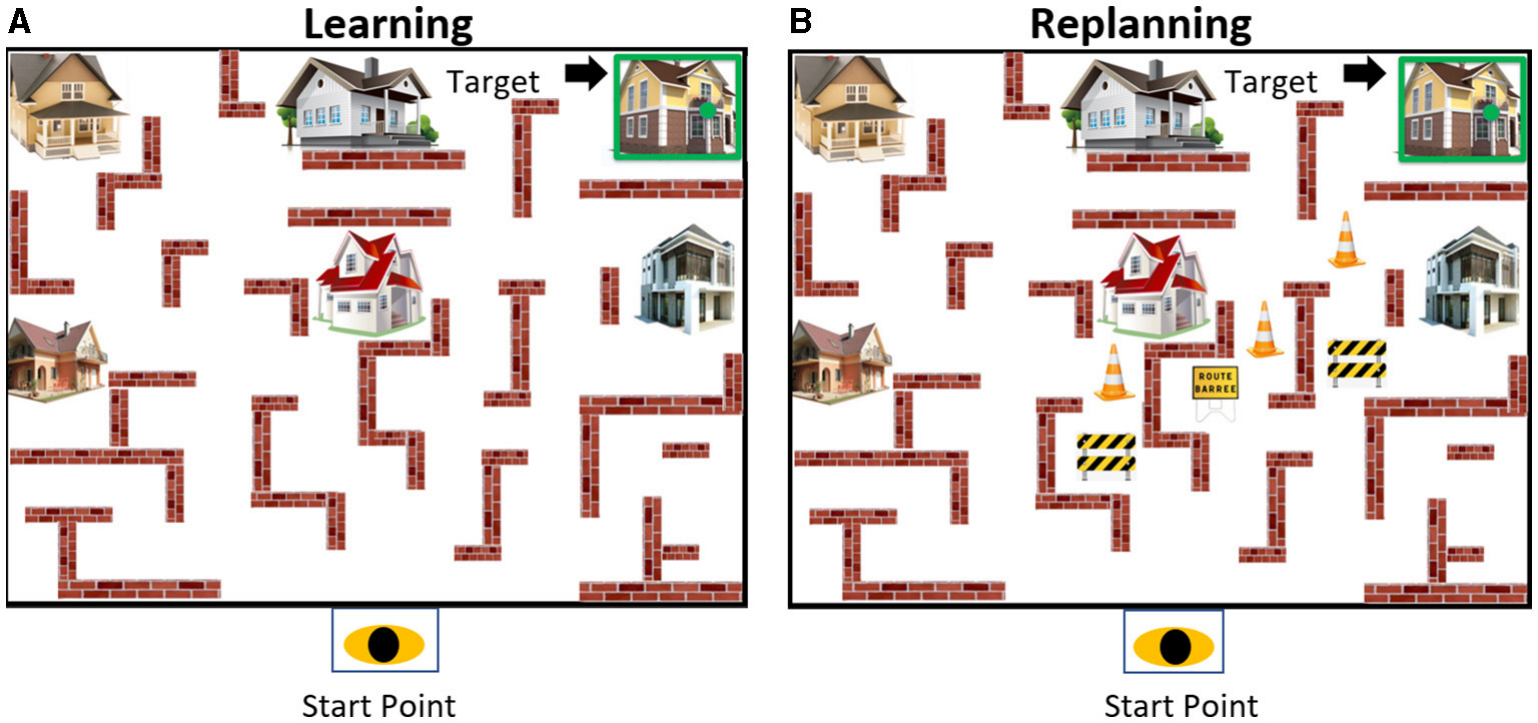
The Virtual Maze layout. **(A)** The left image shows a target house highlighted in green. The same house was shown 5 times for inducing an overlearned trajectory. **(B)** The right image shows a target house highlighted in green and the obstacles blocking access to the house by the shortest paths. This induced the necessity to replan the overlearned trajectory.

With the “Virtual carpet”™, we projected on the floor a virtual maze named the “Virtual House Locomotor Maze” (VHLM™) which allowed us to study the behavior and cognitive control during locomotion. The VHLM™ is composed of 6 houses placed about in a simplified labyrinth delimited by walls created using Microsoft PowerPoint software 2016 (Figure 2). Each house can be identified as a target for the subject by a green dot appearing on the house and surrounding it by a green light square. A beep sound is launched simultaneously to the lighting of the house to increase the attentional focus of subjects on the target house. The projection on the floor delimited the navigational space environment (3.5 x 2.5m). This maze deals with spatial memory of the images of a simplified labyrinth with houses projected on the floor. It requires that the participant generates mental representations of the array, stores them and can recall them. When the participant has to navigate in the virtual labyrinth redirecting himself to the departure point, the process can even engage mental rotation processes (28, 29). This paradigm allows us to also study changes in perspectives during spatial navigation (26). Our paradigm is therefore very adequate for replanning procedures because of the diversity of the brain strategies dealing with space. It offers a large repertoire of choices that participants can use and that we can evaluate in order to understand the basic mechanisms of executive functions and the role of inhibition in the locomotor space.

### Procedure

The experimental design was divided into two assessments: (a) the visual-spatial assessment (VSA) and (b) the goal-oriented locomotion task (GOLT) on the Virtual House Locomotor Maze (VHLM).

#### Visual-Spatial Assessment (VSA)

The visual-spatial assessment was implemented as a control in order to test the visual perception of the stimuli presented in the navigational space and to introduce or familiarize the participant with the VHLM. Every house was presented five times in a random order for a period of 1 s with an interval of 1 s between each stimulus (23, 30). The participant's task was to point to the house that was previously highlighted in green without making any sound. The experimenter supervised whether the participant pointed to the right house in order to include or exclude the participant from the experiment. Following the VSA, the participants performed the goal-oriented locomotion task (GOLT).

#### The Goal-Oriented Locomotion Task (GOLT)

The GOLT was divided into two conditions, (a) the learning condition and (b) the replanning condition where participants are asked to walk on the VHLM to reach the house target presented randomly. In both conditions, the participant always starts each trial at the start point location. The participant was asked to reach a target house highlighted in green accompanied simultaneously by a sound (i.e., beep). They were instructed to reach the target as fast as possible by selecting the shortest path. They were instructed to avoid crossing any walls or obstacles and returning to the start-point while waiting for another trial to begin. Additionally, the participant is also instructed to avoid any possible obstacle that blocks his path to reach a house. The same target house is presented in the learning conditions and in the replanning condition (Figure 2).

##### Learning Condition

In the learning condition, each house is presented five times (i.e., five trials) encouraging the participant to learn and automatize his/her trajectory plan.

##### Replanning Condition

After the learning condition, the participant performs the replanning condition. In this condition, the same target house is presented but the shortest path is permanently blocked. The participant is then required to replan a new trajectory in order to reach the target and return to the start-point.

### Data Acquisition

The Virtual Carpet™ paradigm includes a software (the Basic Trajectory Software (BTS) for recording kinematic information occurring during locomotion. It uses the drives of the HTC Vive: (a) to generate the target positions (i.e., the houses) in the virtual environment known as the calibration procedure, and (b) to record the trajectories of the participant during navigation. This information is saved in two different files: the first file contains the X-axis and the Y-axis coordinates for the locations and the second file contains the participant's locomotion information.

**Figure 3:**
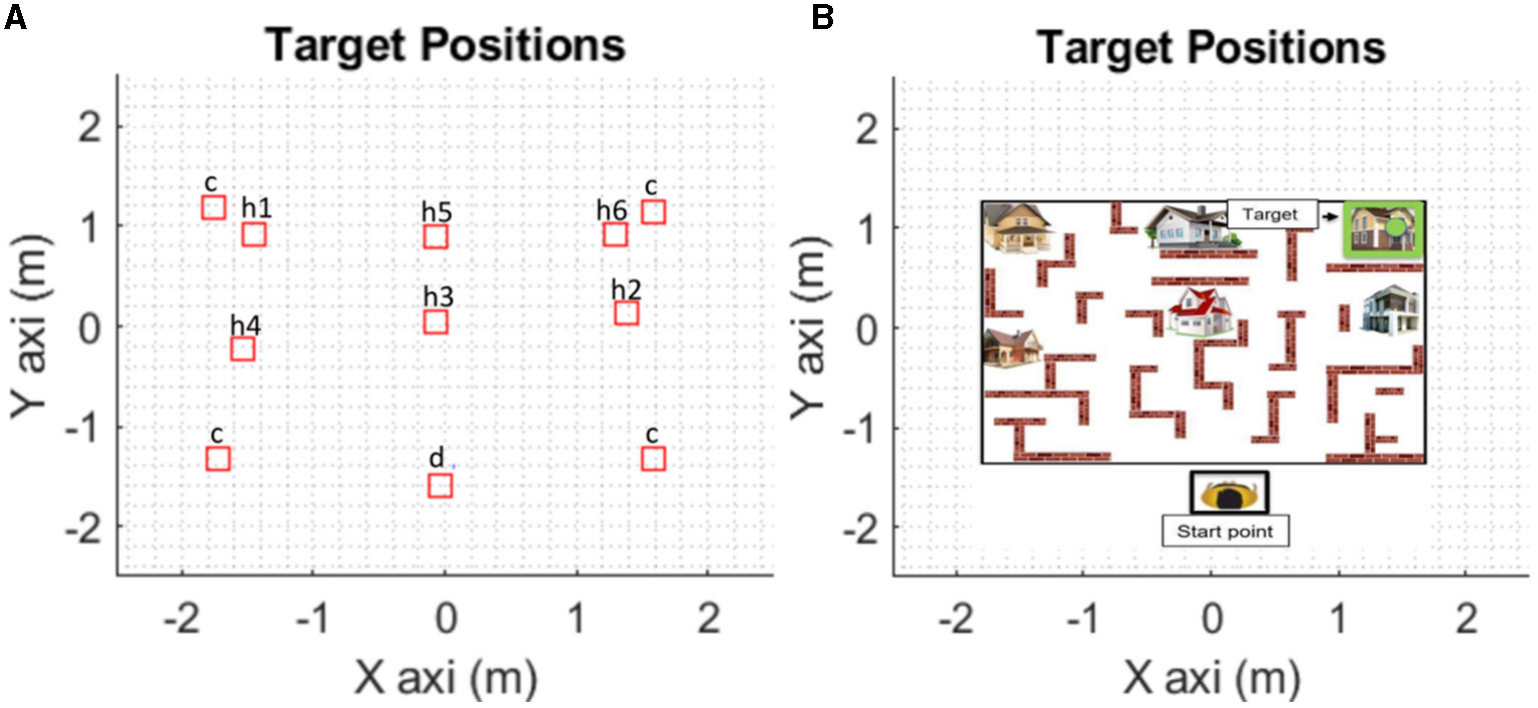
The global navigational array in the X and Y- axis coordinates. **(A)** The “c” represents de corners, the “h1”, “h2”, “h3”, etc. represent the house order presented to the participant and the “d” represents de Start point. **(B)** Representation of the projection on the floor including the houses and the start point.

The calibration procedure enables us: (i) to configure the global navigational array; (ii) to mark the four reference corners of the array; and (iii) to set the target's (houses) positions in a Cartesian coordinate system by triggering the 3D motion sensor (Figure 3). The calibration procedure is performed by the participant by placing himself over each target house following a standard order.

Locomotion is recorded during the experimental sessions using the HTC Vive handheld 3D motion sensors to track the participant navigation. Two HTC cameras detected the handheld 3D motion sensors in the navigational space, which registered their positions. Each trajectory trial is saved individually as a TXT file: column 1 contains the time span for each participant's trail (ms). Time zero corresponds to the moment when the experimenter launched the recording. The columns 2, 3, 6, and 7 represent the participant's head and waist position named PosX, PosY in meters (m). Columns 4, 5, 8 and 9 (i.e., Pitch head and waist and Yaw head and waist) indicate rotation angles with respect to the X and Y- axis direction (Supplementary Table 1). For the purpose of the present study, only the horizontal component of the 3D motions sensors was measured.

The raw data was treated using the Matlab 2019 programming language. Initially, an automatized script read the trajectory file and the targets position file was generated by the BTS. The corporal adjustments (i.e., artifacts) in the standing position at the start point prior to the stimulus presentation were deleted for each trial. The data were filtered using an averaging filter of 2-by-2 neighborhood algorithm. We conducted: (a) a trajectory and head direction qualitative behavioral analysis, and (b) tangential velocity (cm/sec) and latency quantitative analysis.

#### The Trajectory and Head Direction Analysis

**Figure 4:**
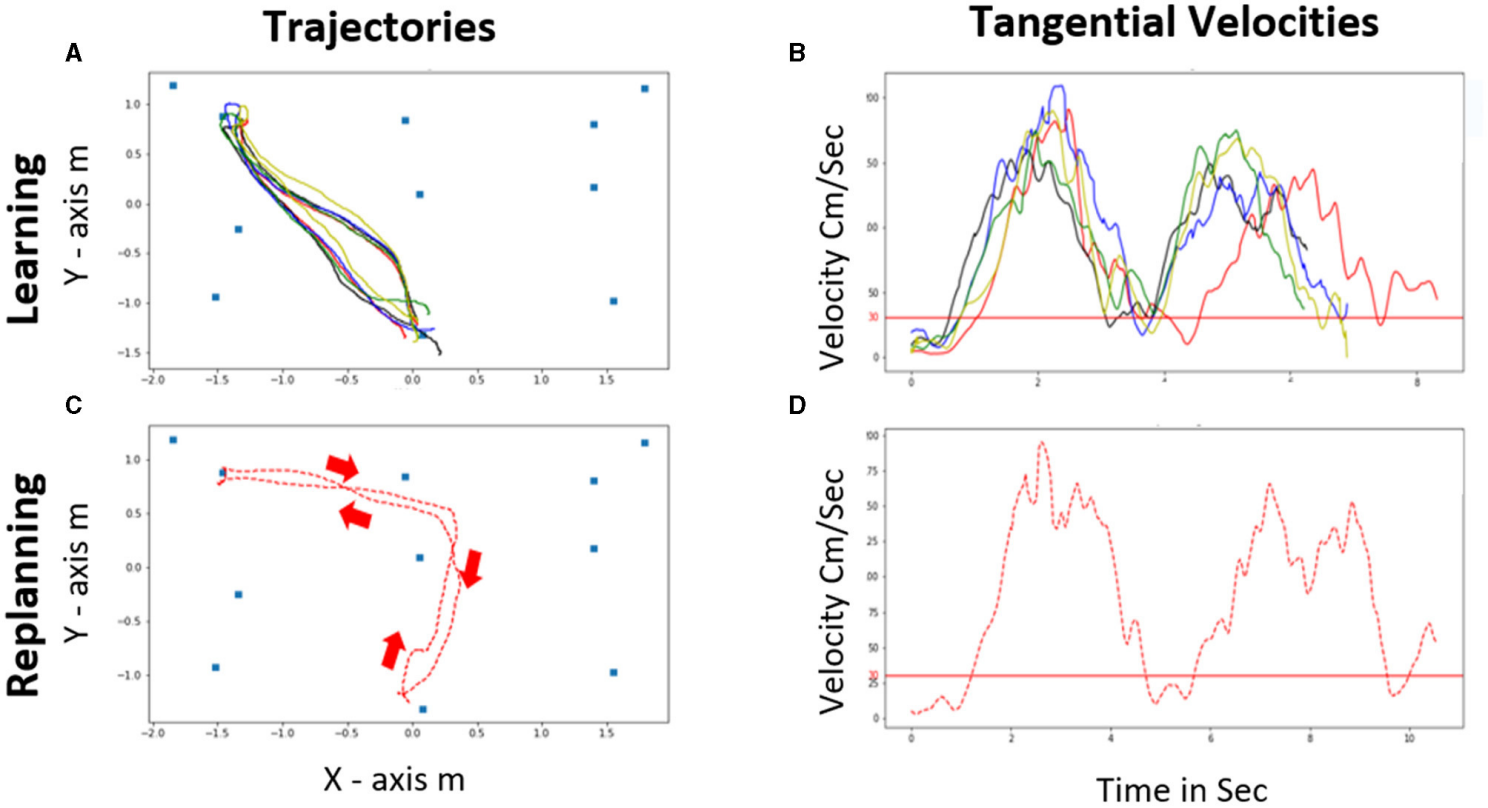
Representation of the Learning and the Replanning phase for a single participant : **(A)** Trajectories and **(B)** tangential velocities for the learning. **(C)** Trajectory and **(D)** tangential velocity for the replanning phase for the first house. The red arrows represent the direction of the trajectories for the replanning phase showing a *new direction* (ND) adapted reconfiguration during the replanning.

By using the trajectory locomotor analysis and the head direction in the horizontal plane, it was possible to identify: (i) the kinematic behavior during the planning and replanning phase, (ii) the behavior during the initial segment of trajectory (Figures 4A,C).

#### The Tangential Velocity (cm/sec)

**Figure 5:**
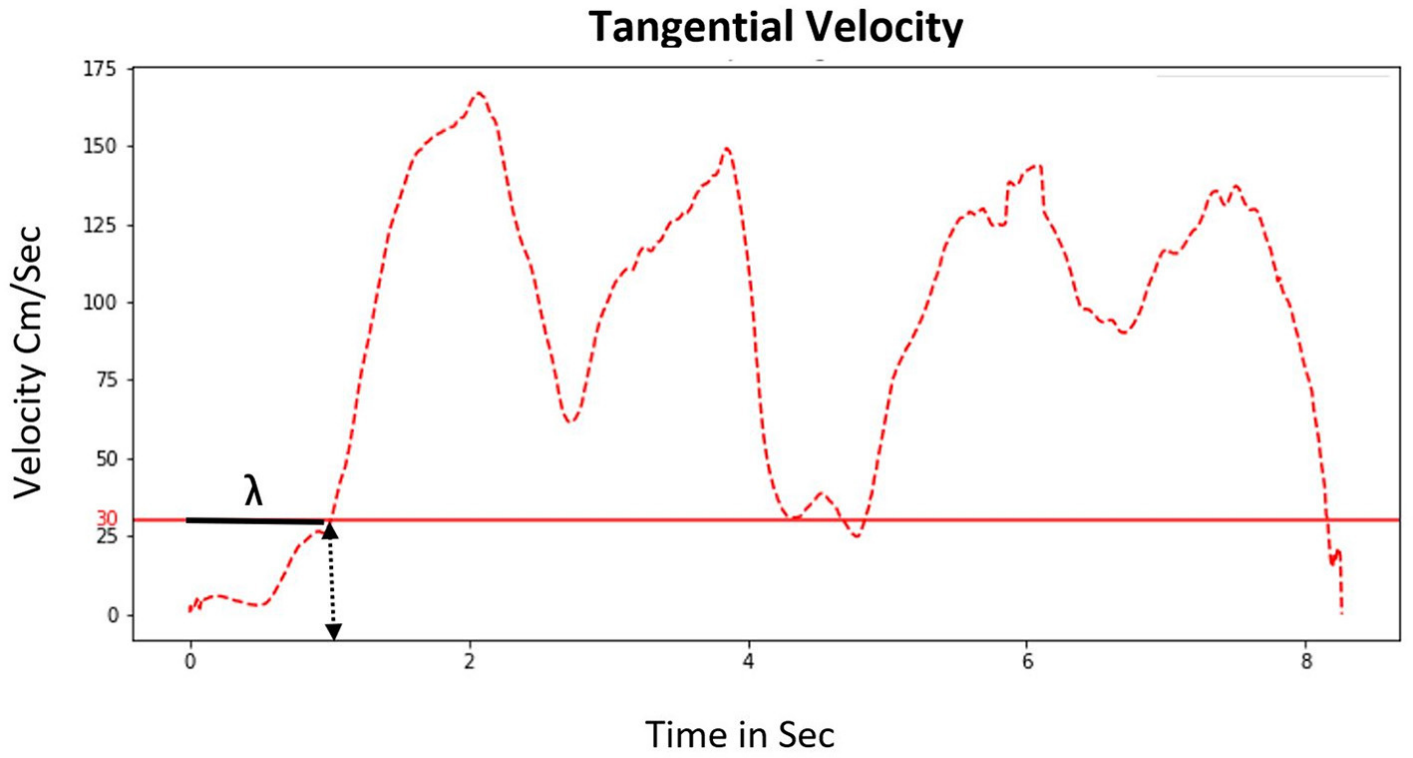
Representation of the computing tangential velocity and latency (Lambda, **λ**) of one participant during a trail in the Learning phase. In the X-axis, the zero corresponds to the moment when the house was lit on the ground indicating “GO” signal. The time of real departure of the subject was computed when the tangential velocity was above the threshold of 30 cm/sec. The “**λ**” is the delay between the “GO” signal and the velocity exceeding the threshold.

It is the velocity produced during the trajectory (see formula below) (Figures 4B,D). Thereafter, the latency or departure time cm/s (DT) was computed from the tangential velocities. The latency corresponds to the delay between the presentation of the target house (i.e., “GO” signal, house lit and beep), the initiation of the locomotor departure and once his tangential velocity reached above the threshold of 30 cm/s (Figure 5). This threshold is arbitrary but considering that standard average walking velocity is 125 cm/s, this represent a 24 % threshold (31).


(1) $$v\left(t\right)=\;\sqrt{\dot{x}{\left(t\right)}^{2\;}+\dot{y}{\left(t\right)}^{2\;}}$$

### Statistical Analysis

All statistical analyses were performed using the open-source statistical software R version 4.0.3. for Windows (www.R-project.org). The collected data were analyzed using descriptive statistics. Given the sample size, these are only exploratory statistics.

## Results

### Tangential Velocities and Latencies for Each Trial

**Figure 6:**
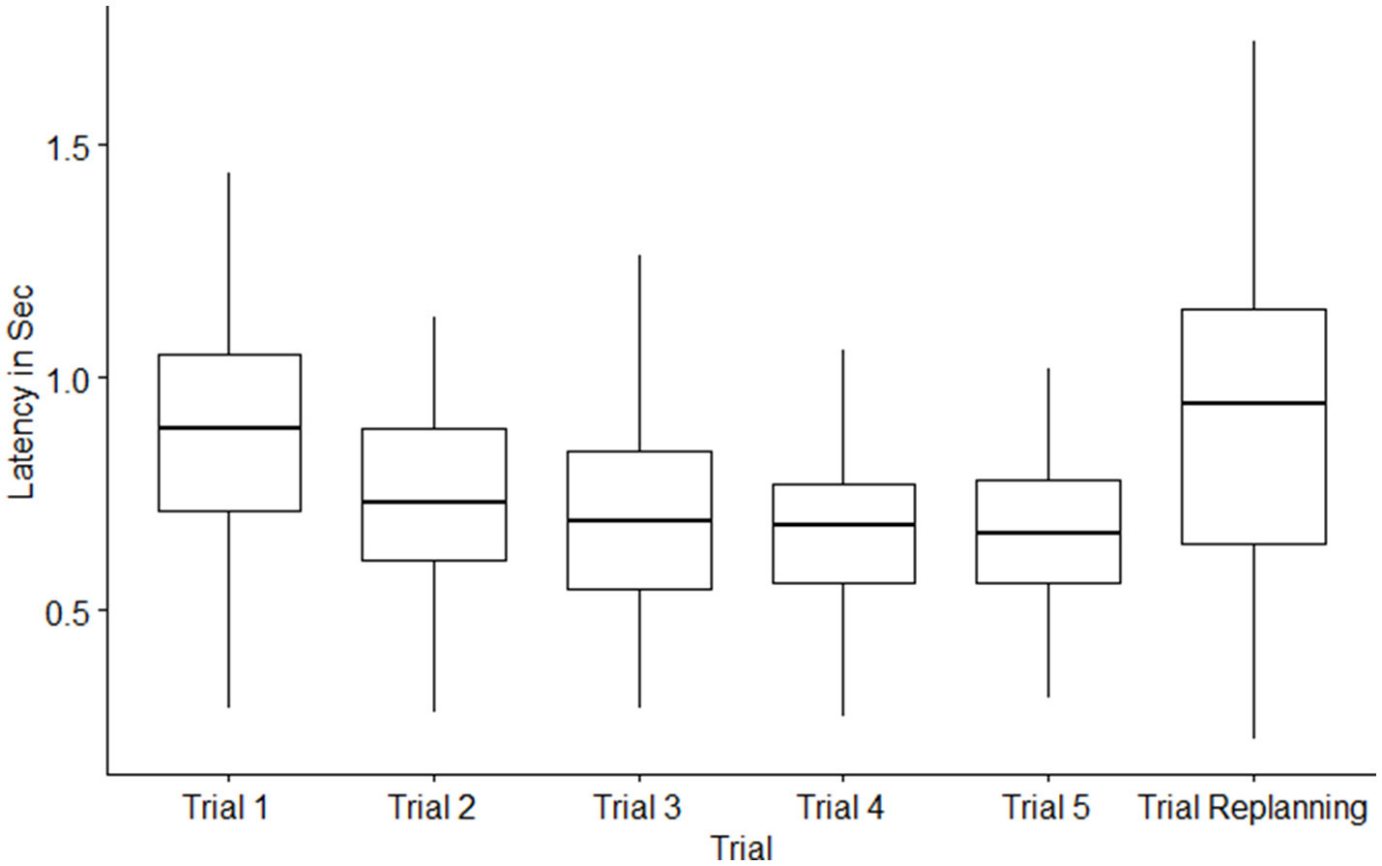
Boxplot of latency in Sec by trials (Leaning and replanning). Box = 25th and 75th Percentiles; bars = min and max values.

The main results are shown in Tables 2, 3 and Figure 6. During the learning phase, from the first trial to the fifth trial the tangential velocities' means increased and it decreased again during the replanning trial. The means of the latencies between target presentation and initiation of locomotion decreased from first trial to the fifth trial. However, the latency increased again during the replanning phase.

**Table 2:** Means (M) and Standard Deviations (SD) of tangential velocity and latency in sec by trials.

	**Tangential Velocity**		**Latency**	
**Trial**	**M**	**SD**	**M**	**SD**
1	81.7	13.6	0.88	0.25
2	85.4	15.5	0.72	0.19
3	87.5	13.0	0.70	0.20
4	89.9	14.2	0.66	0.18
5	90.0	16.7	0.65	0.19
Replanning	82.3	10.9	0.90	0.35

**Table 3:** Median and range quartile (Q1–Q3) of the tangential velocity and the latencies by trials. (*n*) represent the number of observations.

**Dependent** **variable**	**Trial 1** **(*****n*** **= 62)** **median value [Q1;Q3]**	**Trial 2** **(*****n*** **= 56)** **median value [Q1;Q3]**	**Trial 3** **(*****n*** **= 55**) **median value [Q1;Q3]**	**Trial 4** **(*****n*** **= 57)** **median value [Q1;Q3]**	**Trial 5** **(*****n*** **= 58)** **median value [Q1;Q3]**	**Trial** **replanning** **(*****n*** **= 58)** **Median Value [Q1;Q3]**
Tangential Velocity Cm/Sec	81.8 [73.8; 91.0]	84.6 [73.8; 95.5]	86.6 [78.6; 95.8]	86.1 [81.0; 99.8]	88.5 [78.0; 96.1]	83.81 [77.4; 88.1]
Latency (Sec)	0.89 [0.71; 1.04]	0.73 [0.60; 0.89]	0.69 [0.54; 0.84]	0.68 [0.56; 0.77]	0.66 [0.56; 0.78]	0.94 [0.64; 1.14]

### Kinematic Behavior During Learning Phase

**Figure 7:**
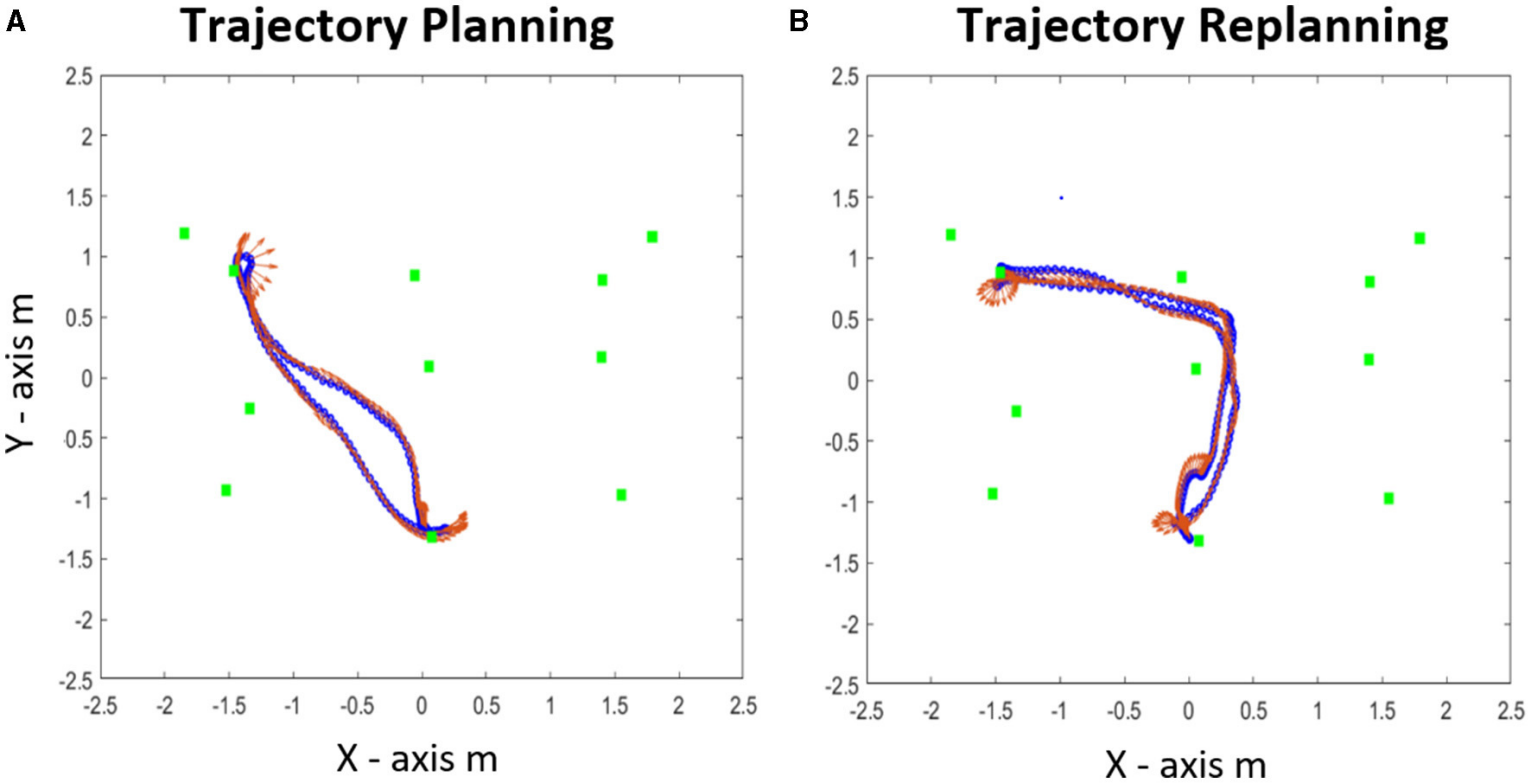
**(A)** An example of a trajectory for the learning phase. **(B)** An example of a trajectory for the replanning phase. The red arrows represent the head direction of the trajectories for each phase.

Figure 7A shows an example of the recorded trajectories and head direction during the learning phase. The blue line represents the head trajectory in the horizontal plane for the head 3D motion-sensor (but a similar graph could be draw for the trunk trajectories). The red arrows indicate the head direction with respect to the experimental room. From this graph, it was possible to identify two behavioral interesting features: (a) overall, subjects went to the target house using the shortest path; (b) once subjects had chosen a trajectory, they generally repeated it with small or rare variations in the trajectories.

### Kinematic Behavior During the Replanning Phase

**Figure 8:**
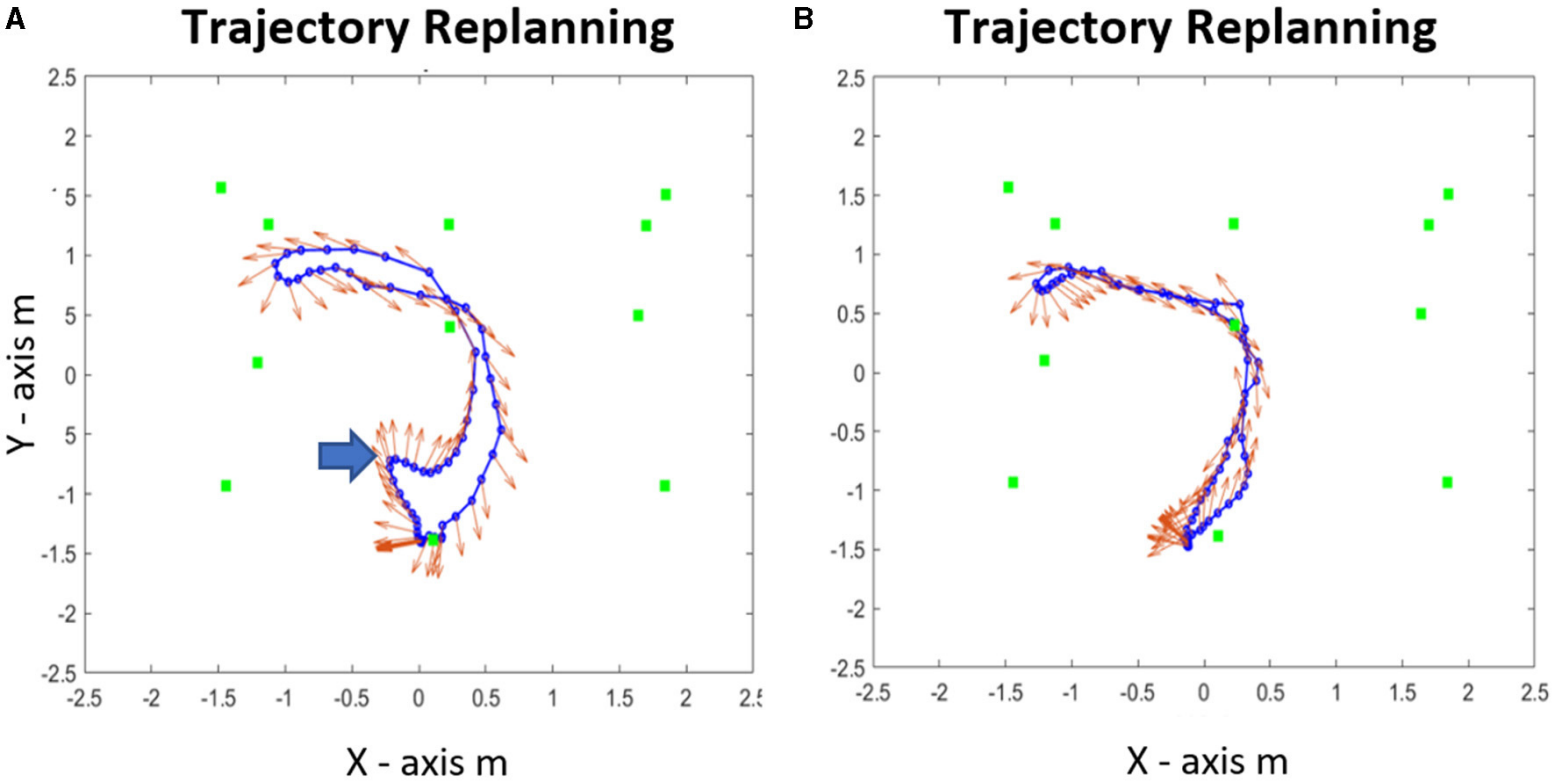
Trajectories replanning examples. **(A)** Departure to *previous learned direction* (PLD) during replanning before the treatment. **(B)** Departure to a *new direction* (ND) after the treatment. The red arrows represent the head direction of the trajectories during the replanning phase.

When the learned path was blocked, as shown on Figure 7B, the subjects chose a new path. We concentrated our analysis on two features of the initial response of the subjects behavior, namely the initial segment of trajectory. Often, the subject started immediately walking in the pre-learned direction (PLD) instead of walking in the direction of a new path (Figure 8A). Then, they could either stop and reorganize the path or continuously redirect the locomotion toward a new goal. In many cases however, the subject would immediately start in the replanned new direction (ND) because they have mentally planned a new trajectory while waiting at the departure point. This can be observed both from the trajectory, and from the head direction vectors in red (Figure 8B). Such a variety of strategies had been observed in a virtual maze paradigm (32). We have named these three behaviors: *the impulsive response with stop, the impulsive response with online planning*, and *the anticipatory- planning*.

### Age Differences

We made two observations concerning the age differences based on the trajectories and the head rotation that suggest further exploration of age differences. Firstly, we found that two very young children (7–8 years old) made a great number of head direction changes during both the learning phase and the replanning phase, and that the oldest subjects did not show this behavior. Secondly, very young children in our sample made more impulsive responses with starts in the wrong direction. It is also know that a progressive maturation of executive functions occurs in the age range of this study from 5 years old to adolescence. Finally, it has been shown that this acquisition of cognitive flexibility is related to the progressive use of inhibition allowing a shift of automatic heuristics to new strategies (6).

## Discussion

The objective of the current study was to design and to test a new protocol named the “Virtual House Locomotor Maze” (VHLM). It allowed us to study cognitive control during navigation throughout neurocognitive development based on the Virtual Carpet paradigm. We explored the capacities of planning and replanning trajectories to study inhibition and mental flexibility using a spatio-temporal index by the means of measuring kinematic behavior (i.e., trajectories, tangential velocity and head direction) during spatial navigation. We concentrated our analysis on : (a) comparing the departure's latency (delays) of the overlearned paths and the new replanned path, (b) analyzing directional locomotor trajectories during replanning, and (c) exploring age and cognitive performance.

During the learning phase, we found a decrease in latency after the repetition of the same target which indicates that there is an ongoing learning and automatization process of the locomotor trajectory. The duration of this initial delay is about 1 s. This delay is compatible with a complex reaction time (about 300 ms), with an additional 600–800 ms time for initiation of gait under cognitive control of a spatial task (33–35). We observed thatbetween the second to the fifth learning trial there is a rapid optimization of the trajectory following the first learning trial throughout the initial trials. In addition, we observed that in the learning condition, the generation of locomotor trajectories follow a kinematic stereotypy (i.e., repetition of the same trajectory) which has already been proven by previous research (36, 37).

The latency (λ) differs between the last (5th) learning trial and the replanning trial by about 0.48 s, reflecting the existence of a delay caused by the inhibition process of the overlearned path. This is observed, in general, when an automatic response (i.e., heuristic) needs to be replaced by a new one with an inhibition process (8). Interestingly, the mean latencies from the first learning trial and the replanning trial are similar, suggesting that the production of a “new” locomotor trajectory engages similar cognitive strategies.

Another interesting observation is the large variability of initiation latencies for the replanning. This variability may reflect the differences of cognitive processes occurring in different subjects depending upon several factors. For example, the encoding of the spatial array of houses may differ according to attentional differences or oculomotor strategies. It is also know from previous work using the magic carpet that children from ages 5 to 12 differ in their use of spatial reference frames (allocentric or egocentric) (26). Also, oculomotor strategies have been found to influence the performance in the planning of a visuo-spatial locomotor task (38).

In our experiment, the learning process is modulated by the task rules, the target spatial location and the temporal constraints (“as fast as possible”). The subject needs to acquire the information about the environment and tries a first path in the virtual labyrinth. This process has been named the “*exploration phase*” (39). In the following trials, this first attempt is used to confirm or modify the “shortest path” chosen by the subject to reach the target house. It has been called the “*exploitation phase*”. A clear distinction of brain activation is identified in the animal model in both phases. The cortical areas and striatum are involved in the *exploration phase* and the hippocampus and cerebellum are involved in the *exploitation phase* (40). In addition, it has been also suggested that the human posterior hippocampus (PH) invigorates exploration while the anterior hippocampus (AH) supports the transition to exploitation on a reinforcement learning task with a spatially structured reward function (41). Thus, our findings are aligned with neuroscience literature suggesting that after repeating the same behavior (i.e., a path), this process induces a shift of neural activity from the prefrontal cortex areas to the basal ganglia cerebellar circuits that “automatizes” the behavior for a faster responses (42, 43). Hence, when the chosen overlearned path is blocked, it will require from the subject to create a new alternative path involving inhibition of the previous path. The prefrontal cortex is reactivated for adapting to this situation and accomplishing the given task (44–46). Thus, prefrontal regions may contribute to spatial navigation involving cognitive processes such as decision-making, goal tracking, and planning (47).

### Strategy Selection and Neurodevelopment

We consider that the combination of locomotor initiation latency and start toward the pre-learned direction should be a good index of impulsivity. Regarding the learning phase, it would be interesting for further research to consider two types of cognitive strategies which could be adopted within the experimental design. The first type of learning relates to a sequential selection of the path (i.e., trial by trial). However, the subject can also anticipate the blocking of the path and adapt their behavior. In addition, an optimal strategy needs to deal with a tradeoff between faster responses and correct responses (for instance, avoiding to go through the virtual walls of the labyrinth) which is a good indicator of cognitive control during neurodevelopment (48).

In the re-planning trial, subjects need to inhibit the overlearned path and re-explore and exploit a new path. We observed that, due to the task's temporal constraints, behaviors can manifest in three different forms:

(a) *Departure without inhibition or impulsive responses:* the subject starts walking toward the target but in the wrong direction (i.e., good target- through the blocked path), then stops and changes the trajectory. We speculate that in this case the subjects are mentally operating in an egocentric frame. Spatial locations are encoded relative to the body (49) without the use of an allocentric global representation of the whole array. Thus, after the blocking of the learned path, the subjects need more time to process spatial information and to plan a new path because they have to shift from the egocentric to the allocentric frame (26).
(b) In the *impulsive online planning*, the subject starts walking toward the target with the learned trajectory and changes the trajectory without completely stopping but instead reduces velocity. In this case, the performance requires a more continuous switching in strategies from egocentric to allocentric treatment. This switching of strategies has been previously reported in children, associated with the maturation of executive functions during the spatial treatment (26). The tangential velocities along the trajectory are somewhat slower as the subject performs on line correction of their trajectory.
(c) In the *anticipatory- planning*, the subject waits at the start point and selects the alternative path avoiding going to the wrong pre-learned direction. In this case, the subject inhibited the automatic response to replan a new trajectory using allocentric encoding before starting the locomotion. It can be associated with longer latencies before the initiation of the locomotion but once a new path has been identified, a higher velocity and amplitude in the tangential velocity is distinguished. We consider that these navigational capacities are acquired throughout child development process and achieved in early adolescence (50).

These results indicated that the VHLM is a suitable tool for assessing visuo-spatial memory, inhibition and cognitive flexibility in the near distant extra-personal locomotor space. The VHLM differs from existing protocols which can assess the behavioral strategies involved during the replanning of a new trajectory by implementing behavioral strategies using navigational arrays. They could also be a good index for development of spatial abilities from childhood to adult.

### Limits and Perspectives

This study has, however, several limits which at the same time encourages further studies: Firstly the small number of subjects could not allow us to jump to conclusions (51). However, we hope that our navigational protocol encourages further extensive research in visuo-spatial memory and replanning including not only children with atypical development such as ADHD and autistic spectrum disorder (ASD) patients but also adults.

Secondly, future research could incorporate new measurements such as the head direction and the difference of head direction with trunk direction (Gaze control Parameter in the absence of eye movements measurements). If a subject who plans, and executes a path with an egocentric sequential set of body movements will make a small number of gaze direction movement. A subject with attentional disorders, or who has difficulties in encoding correctly the array and the location of the target house, or the spatial distribution and geometry of the “streets” in the labyrinth will make more head orientation movement during the replanning phase. For this, it is important to include very light head mounted eye movement devices in order to have precise measurement of gaze direction as was done in Bernardin et and Authié (52–55). However one has to avoid loading the head of children with equipment as this may cause modifications or the behavior.

We will also adapt the negative priming paradigm of O. Houdé and G. Borst group to the VHLM for studying the inhibition process during navigation in typical and atypical development such as ADHD, motor coordination disorder and/or autism spectrum disorder (56). This protocol can also be used to evaluate the contribution of the different action spaces and it is therefore of high interest to better understand complex cases (e.g., multidimensionally impaired) (57).

This paradigm could be used to analyze also non pathological dimensions of personality. For example the possible correlation between different dimensions of the Gregarious Positioning (e.g., Submission and Dominance) (58, 59) and the navigational pattern during the performance in the VHLM. Submission is generally associated with a tendency to remain egocentric and locked on rules and learned behaviors, dominance is generally linked with more global allocentric encoding and an easier capacity to escape rules and find new solutions.

Lastly this protocol could be used in elderly patients, and in particular with Alzheimer patients for testing not only their spatial memory but their spatial cognitive flexibility. It could also be attempted to use is as remediation training for these pathologies.

## Data Availability Statement

The raw data supporting the conclusions of this article will be made available by the authors, without undue reservation.

## Ethics Statement

The studies involving human participants were reviewed and approved by Paris University's ethical committee - 2019-26-CASTILLA-COHEN. Written informed consent to participate in this study was provided by the participants' legal guardian/next of kin. Written informed consent was obtained from the minor(s)' legal guardian/next of kin for the publication of any potentially identifiable images or data included in this article.

## Author Contributions

AB, JF, CL, MZ, and AC designed the study. AC collected and analyzed the data. AB, DC, and AC interpreted the results and wrote the manuscript. OH and GB supervised and helped with the revision. All authors contributed to the article and approved the submitted version.

## Funding

AC was supported by a CDI grant from Institute de Médecine Environmental (IME).

## Conflict of Interest

The authors declare that the research was conducted in the absence of any commercial or financial relationships that could be construed as a potential conflict of interest.

## Publisher's Note

All claims expressed in this article are solely those of the authors and do not necessarily represent those of their affiliated organizations, or those of the publisher, the editors and the reviewers. Any product that may be evaluated in this article, or claim that may be made by its manufacturer, is not guaranteed or endorsed by the publisher.
