# Supplementary material for: A New Paradigm for the Study of Cognitive Flexibility in Children and Adolescents: The “Virtual House Locomotor Maze” (VHLM)
Source: Front Psychiatry. 2021 Sep 23;12:708378. doi: 10.3389/fpsyt.2021.708378 (PMC8495412; doi:10.3389/fpsyt.2021.708378)
Supplement: Supplementary file 1 [file Table_1.DOCX]

**Supplementary Table 1.** A sample of raw data generated by the Basic Trajectory Software version (BTS).

| **Time ms** | **PosX Head m** | **PosY Head m** | **Pitch Head deg** | **Yaw Head deg** | **PosX Waist m** | **PosY Waist m** | **Pitch Waist deg** | **Yaw Waist deg** |
| --- | --- | --- | --- | --- | --- | --- | --- | --- |
| 0 | 0.04 | -1.14 | 11.99 | 352.22 | -0.87 | -1.36 | 346.21 | 307.86 |
| 5 | 0.04 | -1.14 | 12.07 | 352.13 | -0.87 | -1.36 | 346.2 | 307.86 |
| 16 | 0.04 | -1.14 | 12.11 | 352.02 | -0.87 | -1.36 | 346.22 | 307.85 |
| 28 | 0.04 | -1.14 | 12.18 | 351.92 | -0.87 | -1.36 | 346.22 | 307.86 |
| 39 | 0.04 | -1.14 | 12.24 | 351.8 | -0.87 | -1.36 | 346.21 | 307.85 |
| 50 | 0.04 | -1.14 | 12.28 | 351.69 | -0.87 | -1.36 | 346.21 | 307.86 |
| 61 | 0.04 | -1.14 | 12.32 | 351.59 | -0.87 | -1.36 | 346.22 | 307.86 |
| 72 | 0.04 | -1.14 | 12.35 | 351.48 | -0.87 | -1.36 | 346.2 | 307.86 |
| 83 | 0.04 | -1.14 | 12.35 | 351.34 | -0.87 | -1.36 | 346.21 | 307.86 |
| 95 | 0.04 | -1.14 | 12.38 | 351.24 | -0.87 | -1.36 | 346.2 | 307.86 |
| 106 | 0.04 | -1.14 | 12.41 | 351.17 | -0.87 | -1.36 | 346.2 | 307.86 |
| 117 | 0.04 | -1.14 | 12.44 | 351.1 | -0.87 | -1.36 | 346.21 | 307.85 |
| 128 | 0.04 | -1.14 | 12.44 | 351.08 | -0.87 | -1.36 | 346.21 | 307.86 |
| 139 | 0.04 | -1.14 | 12.43 | 351.05 | -0.87 | -1.36 | 346.21 | 307.86 |
