# Supplementary material for: A New Paradigm for the Study of Cognitive Flexibility in Children and Adolescents: The “Virtual House Locomotor Maze” (VHLM)
Source: Front Psychiatry. 2021 Sep 23;12:708378. doi: 10.3389/fpsyt.2021.708378 (PMC8495412; doi:10.3389/fpsyt.2021.708378)
Supplement: Supplementary file 2 [file Data_Sheet_1.pdf]

Annexes ADHD patient

Additionally, we conducted an exploratory test in one patient with ADHD using the VHLM. We have decided to include one ADHD patient in the annexed of this paper to suggest that the paradigm can be also be used with such a patient.

The ADHD patient was a male aged 10 years diagnosed of ADHD and a specific learning disorder (mainly for reading and writing) according to the DSM-V criteria (10). He showed severe learning impairment, impulsivity, lack of attention and hyperactivity. The patient presented an average intelligence with higher verbal abilities according to the Wechsler Intelligence Scale for Children (WISC-IV): Total IQ=111; Verbal IQ=120; Reasoning IQ=116; Working Memory=100; Cognitive speed=88. The reading specialist exam showed normal oral language but very impaired reading skills with phonological (all scores < 1 percentiles), word identification (all scores < 1 percentiles) and spelling (<-1.9 standard deviation) impairments.

The ADHD patient was tested twice: once before taking any medication and once 2 hours after taking the methylphenidate (MPH) treatment prescribed by his psychiatrist. We explored the differences in the replanning performance when the patient was receiving or not a stimulant treatment that is known to limit impulsivity and improve inhibitory control.

We tested the difference in latencies before and after his medical treatment using a Wilcoxon signed rank test. Additionally, we performed a Kruskal-Wallis rank sum test to compare the differences before/after of latencies between trials. Lastly, we tested the differences in the latencies between by trails using Kruskal-Wallis test.

We found a significant effect of the treatment (methylphenidate 20mg per day) on the latencies. The Wilcoxon signed rank test with continuity correction revealed significant differences between latencies before and after the treatment ($p < .004$) with a moderate effect size ($r = 0.478$)**.**The Kruskal-Wallis test did not reveal any significant effects of the trial on the latencies before or after treatment. We did not observe any differences concerning the latencies between trials using the Kruskal-Wallis rank sum test.

We observed a significant difference in the delay caused by the replanning after the blocking of the overlearned path in TD children. As expected, we found a better performance in the reaction time in the ADHD patient when methylphenidate was given. However, it should be noted that we could not, at this stage, distinguish between the effect of the drug and the effect of task repetition i.e., the fact that the same type of protocol was given before and after treatment. For further work on a large number of patients, more research can be done in the department of pedopsychiatry of Hospital Salpétrière (Pr D. Cohen).
